# The Cardiac Acetyl-Lysine Proteome

**DOI:** 10.1371/journal.pone.0067513

**Published:** 2013-07-02

**Authors:** D. Brian Foster, Ting Liu, Jasma Rucker, Robert N. O’Meally, Lauren R. Devine, Robert N. Cole, Brian O’Rourke

**Affiliations:** 1 Division of Cardiology, Department of Medicine, The Johns Hopkins University School of Medicine, Baltimore, Maryland, United States of America; 2 Mass Spectrometry and Proteomics Facility, The Johns Hopkins University School of Medicine, Baltimore, Maryland, United States of America; Federal University of São Paulo (UNIFESP), Escola Paulista de Medicina, Brazil

## Abstract

In the heart, lysine acetylation has been implicated in processes ranging from transcriptional control of pathological remodeling, to cardioprotection arising from caloric restriction. Given the emerging importance of this post-translational modification, we used a proteomic approach to investigate the broader role of lysine acetylation in the heart using a guinea pig model. Briefly, hearts were fractionated into myofilament-, mitochondrial- and cytosol-enriched fractions prior to proteolysis and affinity-enrichment of acetylated peptides. LC-MS/MS analysis identified 1075 acetylated peptides, harboring 994 acetylation sites that map to 240 proteins with a global protein false discovery rate <0.8%. Mitochondrial targets account for 59% of identified proteins and 64% of sites. The majority of the acetyl-proteins are enzymes involved in fatty acid metabolism, oxidative phosphorylation or the TCA cycle. Within the cytosolic fraction, the enzymes of glycolysis, fatty acid synthesis and lipid binding are prominent. Nuclear targets included histones and the transcriptional regulators E1A(p300) and CREB binding protein. Comparison of our dataset with three previous global acetylomic studies uniquely revealed 53 lysine-acetylated proteins. Specifically, newly-identified acetyl-proteins include Ca^2+^-handling proteins, RyR2 and SERCA2, and the myofilament proteins, myosin heavy chain, myosin light chains and subunits of the Troponin complex, among others. These observations were confirmed by anti-acetyl-lysine immunoblotting. In summary, cardiac lysine acetylation may play a role in cardiac substrate selection, bioenergetic performance, and maintenance of redox balance. New sites suggest a host of potential mechanisms by which excitation-contraction coupling may also be modulated.

## Introduction

Acetylation of lysine residues on histones was first recognized as a post-translational modification nearly 50 years ago [1]. In the years since, families of histone acetyltransferases and deacetylases have been discovered, and nuclear protein acetylation has emerged as paramount in chromatin remodeling and transcriptional regulation [2]. The last decade has revealed that lysine acetylation extends beyond the nucleus, ushered by the discovery of a family of NAD^+^-dependent deacetylases. Recently, the advent of new proteomic tools has permitted global scale assessments of lysine acetyl-proteomes [3], [4], [5], [6]. From these studies, it has become apparent that lysine acetylation is a widespread, evolutionarily conserved post-translational modification whose scope rivals phosphorylation.

In cardiac biology, histone acetylation is a mediator of the transcriptional programs that underlie cardiomyocyte proliferation [7], [8], differentiation [9], [10], [11] and cardiac remodeling in pathological hypertrophy (see [12] for a classic review). However, recent work has shown the first glimpses of ways in which non-nuclear lysine acetylation may be at play in the heart. Caloric restriction in mice is cardioprotective and leads to diminished acetylation of mitochondrial proteins, which in turn, correlates with reduced ROS production from the electron transport chain [13]. Others have reported the presence of acetylase/deacetylase activity in the sarcomeres [14], as well as in the gap junctions [15], and a novel mitochondrial lysine acetyltransferase, GCN5L1, has recently been identified [16]. Given the emerging prominence of extra-nuclear lysine acetylation, we undertook a proteomic approach to characterize the broader lysine acetylome of guinea pig hearts under normal physiological conditions. We identified acetyl-proteins unique to the cardiac proteome by mass spectrometry and validated them by immunoblotting.

## Methods

### Animal Care

300 g male Hartley guinea pigs were obtained from Hill Top and housed in an animal facility at The Johns Hopkins University where they had access to a standard chow diet and drinking water *ad libitum*. This study conforms to the Guide for the Care and Use of Laboratory Animals published by the National Institutes of Health (NIH Publication No. 85-23, revised 1996) and was approved by the Johns Hopkins Animal Care and Use Committee.

### Fractionation of the Guinea Pig Heart into Subcellular Fractions

Three guinea pig hearts were isolated and perfused with ice-cold isolation buffer before being minced into pieces about 2–3 mm^3^ in a petri dish containing 5 mL of isolation buffer. The mince for each heart was rinsed twice with isolation buffer and homogenized in isolation buffer supplemented with lysine deacetylase inhibitors, Trichostatin A (1 µM), sirtinol (85 µM), nicotinamide (5 mM), and splitomycin (170 µM), using a chilled Potter-Elvehjem homogenizer (glass tube, Teflon pestle). Homogenates were centrifuged at 700×g for 10 min. The supernatant was set aside and the pellet was resuspended in isolation buffer to extract trapped mitochondria before a second round of centrifugation. The resulting pellet, containing the bulk of the myofilaments, nuclei, and residual mitochondria, was snap-frozen in a dry ice/ethanol bath and frozen immediately at −20**°**C. The supernatants were combined and centrifuged at 8,000×g for 20 min. The pellet containing crude mitochondria and associated plasma membranes and sarcoplasmic reticulum was snap frozen as above. The supernatant was centrifuged at 100,000×g for 1 hr. The pellet and the supernatant (cytosol) were likewise snap frozen and stored at −20**°**C until further use.

### Solubilization and Fractionation of Macromolecular Complexes

Mitochondrial complex I and complex V were enriched from mitochondria by sucrose density gradient as described by Foster *et al*. [17], based on the original protocol by Hansen et al. [18]. Briefly, mitochondria (5 mg protein/mL) were solubilized in ice-cold phosphate-buffered saline (Gibco) containing lauryl maltoside (1% w/v) and supplemented with deacetylase inhibitors (Trichostatin (1 µM), splitomycin (170 µM) and nicotinamide (5 mM)) Residual particulate matter was removed by centrifugation at 72,000×g for 30 minutes at 4°C. The samples were layered on top of a discontinuous sucrose gradient consisting of 1.5 mL layers, starting with 32.5% (w/v) sucrose, buffered with 50 mM Tris-HCl pH 7.5, on the bottom and successive layers of descending sucrose concentration (30%, 27.5%, 25%, 22.5%, 20%, 17.5%). Samples were centrifuged at 132,000×g (r_ave_) in a SW41-Ti rotor for 18 hours at 4°C to resolve the mitochondrial respiratory complexes. Samples were recovered by puncturing the bottom of the polyallomer centrifuge tube with a 23-gauge needle and collecting 1.5 mL aliquots, drop-wise.

### In-solution Digestion of Proteins and Enrichment of lysine-acetylated Peptides

Twelve samples, representing the 4 subcellular components from each heart, were delipidated and precipitated and concentrated by methanol/water/chloroform precipitation as described Wessel and Flugge [19]. Trace organic solvent was removed by streaming N_2_ gas over the samples for 1–2 minutes. The precipitated proteins were re-dissolved in 10 mM HEPES, 6 M urea, 2 M thiourea, pH 8.0. After 4-fold dilution in deionized water such that the final concentrations of urea and thiourea were 1.5 M and 0.5 M respectively [3], samples were digested with modified sequencing grade porcine trypsin (1∶200 w/w). Peptides were reduced with 20 mM dithiothreitol for 30 min, and alkylated with 100 mM iodoacetamide for 1 hr, in the dark. Alkylated peptides were acidified with 0.5% trifluoroacetic acid (TFA) and applied to reversed-phase SepPak C18 cartridges (Waters). Peptides were eluted using 0.1% TFA, 60% acetonitrile. The eluates were evaporated in a Vaccufuge (Eppendorf) until dry. Samples were redissolved in 50 mM HEPES pH 7.2, 50 mM NaCl, 10 mM Na_2_HPO_4_. Immunoprecipitation was carried out as essentially described by Choudhary and colleagues [3] with minor changes. Guinea pig heart subfractions were incubated with agarose-conjugated anti-acetyl-lysine antibody (ImmuneChem) for 24 hrs at 4**°**C on a rotation wheel. The immunoprecipitates were washed 4 times with the immunoprecipitation buffer followed by two washes with distilled water. Residual water was removed and acetylated peptides bound to antibodies were eluted in 0.1% formic acid.

### LC-MS/MS Analysis

Peptides were injected onto a 2 cm trap column at 5 µL/minute for 6 minutes before being eluted onto a 75 µm×15 cm in house packed column (Michrom Magic C18AQ, 5 µm 100A) using a nanoAquity nanoLC system (Waters) operating at 300 nL/min. Each sample was run on a 90-minute gradient with double sawtooth cleanup gradients between each run. The peptides were eluted and ionized into an Orbitrap Velos mass spectrometer (Thermo Fisher) at 2.0 kV using a data-dependent “Top 20” method operating in FT-IT parallel acquisition mode. The survey full-scan MS (m/z from 350–1700) was performed at a resolution 60,000 with a target of 1×10^6^, while the ion trap MS^2^ scans were performed at a target value of 10,000 ions. Maximum injection times were both set to 100 ms. The ion selection threshold was set to 2,000 counts and an isolation width of 1.9 daltons was used to perform CID fragmentation with a normalized collision energy of 35%. Ambient polysiloxane produced a background peak at 371.101230 m/z, which was used as an internal calibrant for each survey scan.

### Database Search Parameters

Peak list files (.RAW) were searched against a guinea pig database of predicted proteins (Ensembl CavPor3_59.pep.fasta; 19744 sequences), using Mascot Version: 2.2.0 (Matrix Science). Spectra were searched with a mass tolerance of 15 ppm in MS mode and 0.8 Da in MS/MS mode. Trypsin was specified as the enzyme and 4 missed cleavage sites were allowed. Cysteine carbamidomethylation was searched as a fixed modification, whereas N-pyroglutamine, oxidized methionine and acetylation of lysine was searched as variable modification. All searches were conducted with the reversed-database search mode engaged. Mascot output files (.dat) were imported into Scaffold 3Q+ (v. 3.1.2), where spectra were also searched against the same guinea pig database, using identical search parameters, with X!Tandem [20].

### Statistical Validation Peptides, Proteins & Acetylation Sites: Acceptance Criteria

Peptide identification probability was assessed by PeptideProphet [21] in conjunction with a high mass accuracy parent-ion scoring model, while protein identification probability was assessed by ProteinProphet [22], as implemented in Scaffold 3Q+ (v. 3.1.2). Peptide data were kept for further analysis if they contained at least one high confidence acetyl-peptide/spectrum assignment (>90% confidence) and the confidence level of the protein to which it mapped was >90%. At these Peptide and ProteinProphet thresholds, the global false discovery rates for this study, obtained from a search of the reversed guinea pig database, were estimated to be on the order of <0.1% and <0.8% at the peptide and protein levels respectively. Proteins identified on the basis of single spectrum/peptide matches were inspected manually and accepted only if they: 1) were well fragmented, and displayed contiguous b- and y-ion stretches, 2) showed complementary b- and y-ions, and 3) conformed with well-established peptide fragmentation biases [23] (e.g. intense ion intensities N-terminal to P, favored fragmentation on the N-terminal side of G or S, and favored fragmentation to the C-terminal side of the branched chained amino acids V, I or L). Data from Scaffold 3Q+ (.mzid) were exported to Scaffold PTM (v. 1.0), where the position of acetylation sites was evaluated and assigned with a statistical probability using the A-score algorithm adopted from Beausoleil *et al*. [24].

### Numbering of Acetylation Sites

To facilitate comparisons with other datasets, the locations of acetylation sites within the guinea putative pig primary sequences were mapped to the corresponding position within human homologs, using the Basic Local Alignment Sequence Tool, blastp (http://blast.ncbi.nlm.nih.gov/Blast.cgi).

### Bioinformatic Analysis

Ensembl protein ID accession numbers were mapped back to their associated encoding Ensembl gene entries, which have been provisionally annotated to Human Genome consortium Gene Names. Gene Ontology annotation of broad Cellular Components (Table 1) was obtained from the Ensembl Gene annotation. More detailed gene-set enrichment analysis was performed using BINGO [25], a Cytoscape [26] plugin. Briefly, gene names were uploaded from Table 1, and analyzed with default parameters, which specify a Benjamini-Hochberg correction for multiple-hypothesis testing, at a false discovery rate of 5%. Since BINGO did not have a guinea pig ontology set at the time of analysis, output Tables and GO-networks were generated using murine GO terms, and limited to terms with p-values <0.01.

**Table 1:** Top 40 Most Heavily Acetylated Proteins in the Heart (by Site Count).

Cell Compartment	Process	Ensembl Proteins	Gene		Protein description	Sites		
Sarcomere	Myofilament	ENSCPOP00000006354*	MYH7	Beta-Myosin Heavy Chain.		49		
Extcell	Lipid Binding	ENSCPOP00000002782*	ALB	Preproalbumin Precursor.		25		
Mitochondrion	Mito FAO	ENSCPOP00000005365*	HADHA	Hydroxyacyl-CoA Dehydrogenase/3-ketoacyl-coa Thiolase/Enoyl-coa Hydratase (Trifunctional Protein), Alpha Subunit		25		
Mitochondrion	Mito TCA	ENSCPOP00000007630*	IDH2	Isocitrate Dehydrogenase 2 (NADP+), Mitochondrial		23		
Mitochondrion	Mito TCA	ENSCPOP00000008228*	GOT2	Glutamic-oxaloacetic Transaminase 2, Mitochondrial (Aspartate Aminotransferase 2)		19		
Mitochondrion	Chaperone	ENSCPOP00000016987*	HSPD1	Heat Shock 60 kda Protein 1 (Chaperonin)		17		
Mitochondrion	Mito TCA	ENSCPOP00000004776*	DLD	Dihydrolipoamide Dehydrogenase		15		
Cytoplasm	Phosphotransfer	ENSCPOP00000016471*	CKM	Creatine Kinase, Muscle		15		
Mitochondrion	Mito TCA	ENSCPOP00000019095*	MDH2	Malate Dehydrogenase (EC 1.1.1.37) (Fragment).		15		
Mitochondrion	Redox	ENSCPOP00000002046*	NNT	Nicotinamide Nucleotide Transhydrogenase		13		
Mitochondrion	Mito Oxphos	ENSCPOP00000003628*	ATP5H	ATP Synthase, H+ Transporting, Mitochondrial Fo Complex, Subunit D		13		
Mitochondrion	Mito Oxphos	ENSCPOP00000004744*	SLC25A4	Solute Carrier Family 25 (Mitochondrial Carrier; Adenine Nucleotide Translocator), Member 4		13		
Mitochondrion	Mito FAO	ENSCPOP00000004877*	HADHB	Hydroxyacyl-CoA Dehydrogenase/3-ketoacyl-coa Thiolase/Enoyl-coa Hydratase (Trifunctional Protein), Beta Subunit		13		
Mitochondrion	Mito Oxphos	ENSCPOP00000011443*	ATP5A1	ATP Synthase, H+ Transporting, Mitochondrial F1 Complex, Alpha Subunit 1, Cardiac Muscle		12		
Mitochondrion	Mito Oxphos	ENSCPOP00000009450*	ATP5C1	ATP Synthase, H+ Transporting, Mitochondrial F1 Complex, Gamma Polypeptide 1		11		
Mitochondrion	Mito TCA	ENSCPOP00000011373*	ACO2	Aconitase 2, Mitochondrial		11		
Mitochondrion	Chaperone	ENSCPOP00000002148*	HSPA9	Heat Shock 70kda Protein 9 (Mortalin)		10		
Mitochondrion	Mito FAO	ENSCPOP00000004266*	ACAA2	Acetyl-CoA Acyltransferase 2		10		
Nucleus	Histone	ENSCPOP00000015130*	HIST1H2BE	Novel Transcript [Type: Protein Coding Ensembl]		10		
Cytoplasm	O2 Carrying	ENSCPOP00000001378*	HBB	Hemoglobin Subunit Beta (Hemoglobin Beta Chain) (Beta-globin).		9		
Nucleus	Transcription	ENSCPOP00000004894*	EP300	E1A Binding Protein P300		9		
Mitochondrion	Mito Oxphos	ENSCPOP00000006024*	UQCRC2	Ubiquinol-cytochrome C Reductase Core Protein II		9		
Mitochondrion	Mito Oxphos	ENSCPOP00000008014*	SDHA	Succinate Dehydrogenase Complex Subunit A.		9		
Mitochondrion	Mito TCA	ENSCPOP00000008332*	PDHA1	Pyruvate Dehydrogenase E1 Component Subunit Alpha Somatic Form MitochondrialPrecursor Ec_1.2.4.1 Pdhe1a Type I		9		
Mitochondrion	Mito FAO	ENSCPOP00000008856*	ACADL	Acyl-CoA Dehydrogenase, Long Chain		9		
Mitochondrion	Mito Ion	ENSCPOP00000009440*	VDAC3	Voltage-dependent Anion Channel 3		9		
Mitochondrion	Mito FAO	ENSCPOP00000010349*	CPT2	Carnitine Palmitoyltransferase 2		9		
Mitochondrion	Mito FAO	ENSCPOP00000013564*	ACAT1	Acetyl-CoA Acetyltransferase 1		9		
Mitochondrion	Mito FAO	ENSCPOP00000015742*	HADH	Hydroxyacyl-coa Dehydrogenase		9		
Nucleus	Histone	ENSCPOP00000016731*	HIST2H2BF	Histone Cluster 2, H2bf		9		
Mitochondrion	Proteolysis	ENSCPOP00000010265*	C21orf33	ES1 Mitochondrial Precursor		8		
Mitochondrion	Mito Oxphos	ENSCPOP00000012673*	ATP5F1	ATP Synthase, H+ Transporting, Mitochondrial Fo Complex, Subunit B1		8		
Mitochondrion	Mito FAO	ENSCPOP00000014862*	ACADVL	Acyl-coa Dehydrogenase, Very Long Chain		8		
Sarcomere	Myofilament	ENSCPOP00000016138	TNNI3	Troponin I Type 3 (Cardiac)		8		
Mitochondrion	Redox	ENSCPOP00000000012*	ALDH6A1	Aldehyde Dehydrogenase 6 Family, Member A1		7		
Mitochondrion	Mito TCA	ENSCPOP00000002733*	SUCLG1	Succinate-CoA Ligase, Alpha Subunit		7		
Nucleus	Histone	ENSCPOP00000004239*	H3F3A	Histone H3		7		
Cytoplasm	O2 Carrying	ENSCPOP00000006188*	MB	Myoglobin		7		
Mitochondrion	Mito FAS	ENSCPOP00000011954*	ACSF2	Acyl-CoA Synthetase Family Member 2		7		
Mitochondrion	Mito Oxphos	ENSCPOP00000016071*	ATP5B	ATP Synthase Subunit Beta (EC 3.6.3.14) (Fragment).		7		

Top 40 Most Heavily Acetylated Proteins (by Number of Acetyl-Lysine Residues). Asterisks (*) designate the accessions of acetyl-proteins identified in all three biological replicates.

### Immunoblot Analysis

Samples were fractionated by gel electrophoresis using 4–12% NuPAGE Bis-Tris gels (Invitrogen) at 150 V for 35 mins (MES buffer) or 50 mins (MOPS buffer). Proteins were transferred to nitrocellulose with the i-Blot system (Invitrogen) using a 9-minute transfer. Blots were blocked with 5% (w/v) bovine serum albumin (BSA) (Cell Signaling Technologies cat# 9998) in Tris-buffered saline containing 0.1% (v/v) Tween-20 for a minimum of 1 hour. Primary anti-acetylated lysine antibodies (polyclonal, Abcam cat#: ab21623; monoclonal, Cell Signaling Technologies cat#: 9681), were incubated in Tris-buffered saline (20mM Tris pH 7.4, 154 mM NaCl) supplemented with 0.1% (v/v) Tween-20 (TBS-T) containing 1% (w/v) BSA overnight at 4°C. Control blots were conducted by preincubating acetyl-lysine antibodies with acetylated BSA (>2% w/v; Sigma cat#:B2518 or prepared in-house according the method of Fraenkel-Conrat [27] as summarized by Riordan and Vallee [28]) for a minimum of 1 hour at room temperature, with agitation, prior to applying the antibody to the blots. Horseradish peroxidase-conjugated secondary antibodies were incubated for 1 hour. After washing extensively with TBS-T, blots were developed using Pierce West-Pico or West-Femto chemiluminescent reagent and imaged on Kodak X-Omat film.

### Structural Modeling of Guinea Pig Protein Sequences

Structural models were generated using the web-based modeling suite, Swiss-Model (www.swissmodel.expasy.org) [29], [30], [31], [32]. Sequences for the N-terminal 838 amino acids of guinea pig myosin heavy chain beta (MHC-β; ENSCPOP00000006354) and the full sequence of guinea pig SERCA2 (ENSCPOP00000002487) were submitted in “Automated Mode”. Guinea pig myosin was modeled on the crystal structure of human MHC-β (PDB ID: 4db1B, unpublished) The guinea pig SERCA sequence was modeled on the rabbit crystal structure (PDB ID: 3AR4; with bound ATP, no Ca^2+^) [33].

## Results

### Specificity and Reproducibility

**Figure 1:**
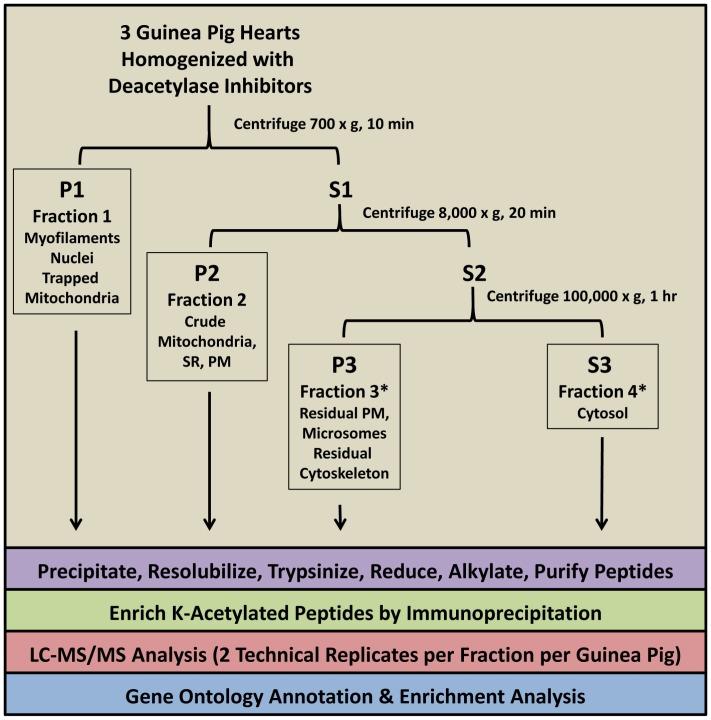
Proteomic Work-flow. Asterisks denote that since the data from the microsomal and plasma membrane fraction yielded few new sites or proteins, it was combined with data from the cytosolic fraction for the purposes of discussion.

To assess the guinea pig proteome, homogenates from 3 guinea pig hearts were fractionated to enrich cellular components and analyzed according to the workflow depicted in Figure 1. Specifically, low speed centrifugation yielded a myofilament and nucleus-enriched fraction (hereafter known as myofilament rich). A crude mitochondrial fraction was obtained by keeping the supernatant from low-speed centrifugation as well as an additional homogenization of the myofilaments to release trapped mitochondria, pooling the two supernatants and collecting the pellet following the 8,000×g centrifugation. The remaining supernatant was centrifuged at 100,000×g to remove residual insoluble material and yield a pure cytosolic fraction. Since few new proteins were found in the insoluble material, however, for the purposes of analysis the data from the two fractions is combined and designated the cytosol-rich fraction.

Following tryptic digest of the subcellular compartments, acetylated peptides were enriched from bulk peptides by immunoprecipitation with immobilized acetyl-lysine antibody as described previously [3], [4], [6]. Subsequent LC-MS/MS analysis initially identified a total of 608 proteins. Of these, 252 (41%) contained acetylated peptides. This contrasted with a preliminary analysis of a crude mitochondrial fraction without acetyl-peptide enrichment, in which only 0.7% of all identified proteins contained acetylated peptides (data not shown). Subsequent manual curation, as detailed in methods, pared 12 putative acetyl proteins identified on the basis of single acetylated peptides from the list. After curation, we identified a total of 994 acetylation sites, from 1075 acetylated peptides that map to 240 proteins and protein clusters. Site localization probability within the acetylated peptides was assessed with Scaffold PTM’s implementation of the A-score [34]. Acetylation at 905 out of 994 sites was ascertained with a probability >0.99; 15 fell between 0.9–0.99 and another 14 between 0.8–0.9. The remainder, 60/994 (6%), had probabilities below 0.8. Just over half of the lower scoring sites (34 of 994 total) came from single spectrum/peptide matches and are best considered with caution (Table S1).

**Figure 2:**
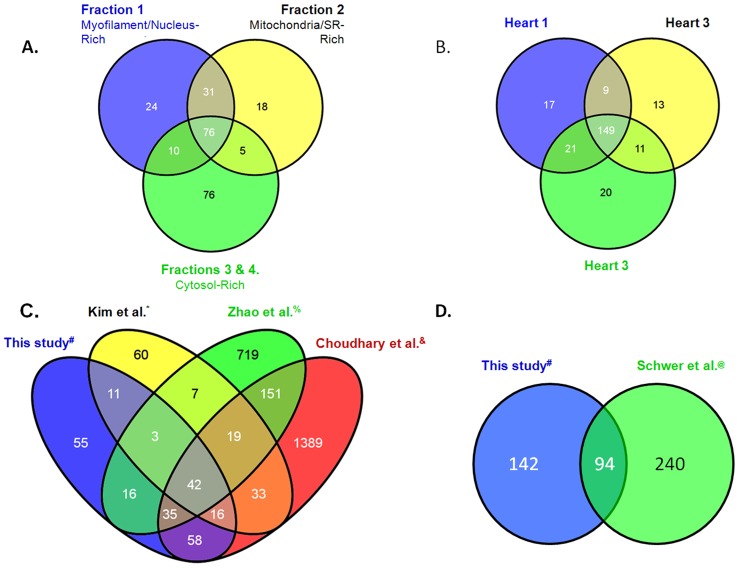
The Dataset. Panel A. Distribution of acetylated proteins by experimental subfraction. Panel B. Distribution by biological replicate. Panel C. Comparison with published global-scale mammalian lysine acetylomes including: (*) mouse liver [4], (^&^) human acute myeloid leukemia cell line, MV4-11 [3], and (^%^) human liver [6]. Panel D. Comparison with the (@) mouse liver mitochondrial lysine acetylome of Schwer *et al* [35].

In concordance with the crude level of subcellular enrichment, there was substantial overlap of identified proteins and acetylation sites between the mitochondrial, myofilament and cytosol-rich fractions (Figure 2A). Of the three experimental cellular compartments, the cytosol-rich fractions, largely free of myofilament and mitochondrial contamination, yielded the most distinct proteome. The reproducibility of the acetylome was addressed by analyzing 3 hearts separately, and by analyzing each subcellular component with technical replicates. 149 acetyl-proteins were found in all 3 guinea pig hearts (62%; Figure 2B). Fully 79% of acetyl-proteins were identified in 2 out of 3 hearts. Approximately 21% of proteins were identified in only one heart, though often by many peptides and spectra. A detailed delineation of the acetyl-peptide and protein distribution is provided in Table S1 (Panel 1, columns G to AO). Comparison of our heart dataset against previous proteomic assessments of global lysine acetylation in both liver [4], [6] and human cell lines [3], showed substantial overlap of genes encoding acetyl-proteins (Figure 2C). Shwer *et al*. also conducted proteomic analysis of isolated liver mitochondria [35], with which our dataset shows considerable overlap (Figure 2D).

### Functional Classification and Gene Set Enrichment of K-Acetylated Proteins

**Figure 3:**
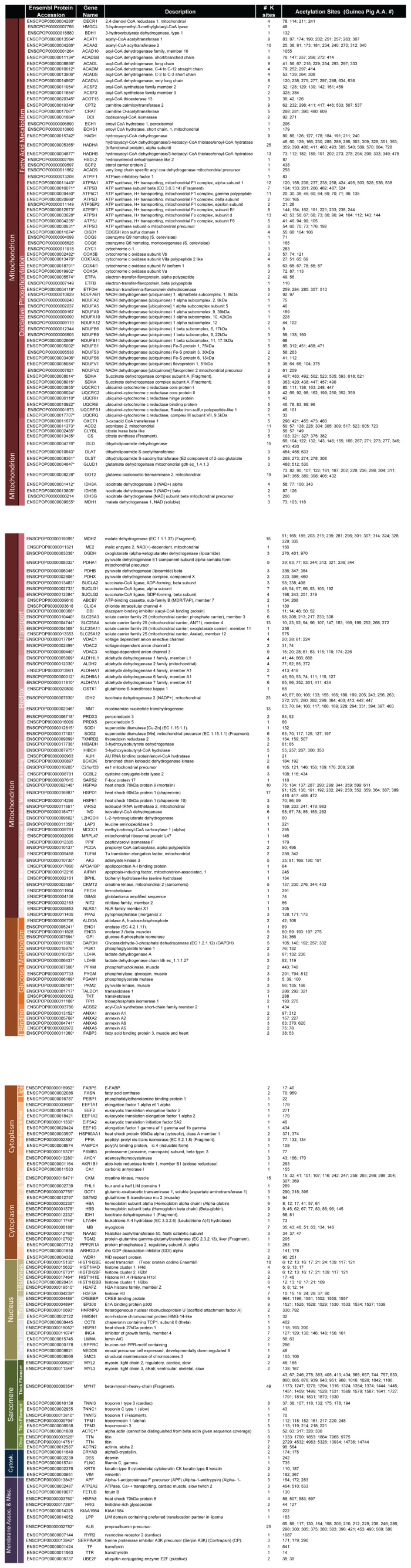
The Cardiac Lysine Acetylome. Acetyl-proteins are grouped first by cellular component and then by molecular function. Asterisks (*) designate acetyl-proteins found in all three biological replicates. Acetylation sites are numbered according to guinea pig sequence. The numbering of orthologous human lysine residues generally tracks very closely with guinea pig sequences. Orthologous human lysines are found in Table S2.

The acetylated proteins and the sites of acetylation are presented in tabulated form in Figure 3. For clarity, proteins have been grouped firstly according to their primary cellular location and then loosely by the biological processes they perform. The number of sites and site location within each protein are also provided. Sites are numbered according to the guinea pig database. However, we have mapped the guinea pig sites to their corresponding lysine residues in humans (Uniprot/SwissProt numbering; Table S2) of which 94% are conserved. Figure 3 shows that 142 of 240 (59%) can be mapped, through their gene ontology, to mitochondrial annotations. Other cellular compartments represented in Figure 3 include the cytoplasm, the nucleus, the sarcomere and cytoskeleton, and finally assorted membrane or membrane associated proteins including proteins of the sarcoplasmic reticulum.

**Figure 4:**
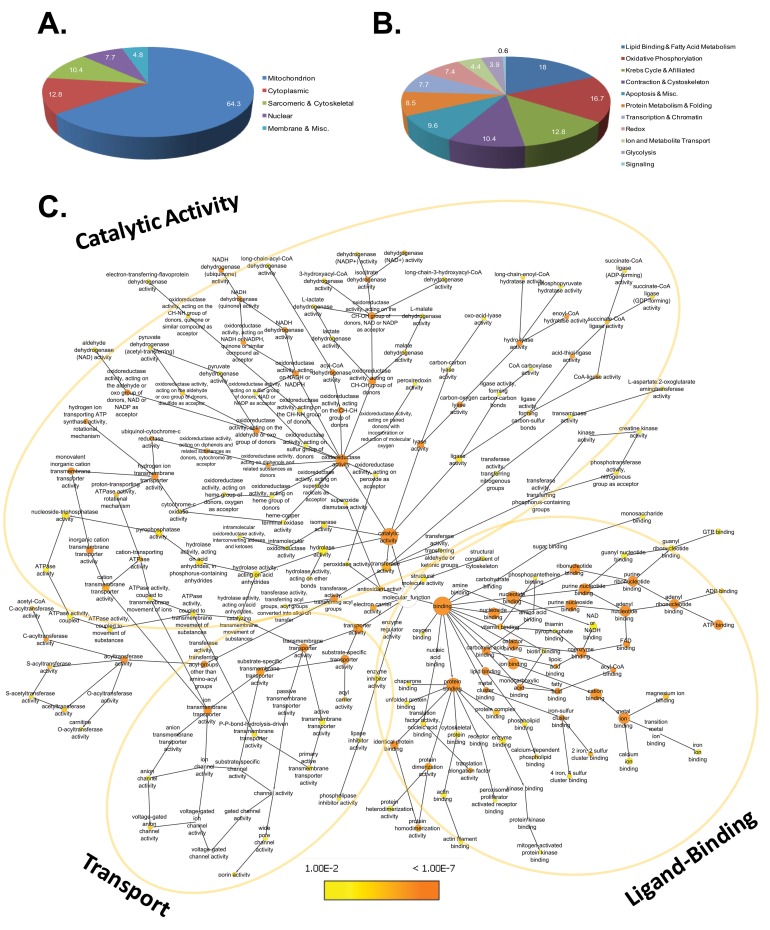
Functional Annotation and Enrichment analysis. Panel A. Distribution of acetylation sites by cellular component. Panel B. Distribution of acetylation sites by biological process. Data for panels A and B were taken from Figure 3 and expressed as percentages of the total number of sites. Panel C. Network of gene ontology annotations for molecular function. Node color represents the magnitude of corrected p-value, a continuous variable that provides a measure of enrichment (see legend). Node size is proportional to the number of genes associated with each GO-term. The layout is broadly grouped by similar molecular functions, including catalytic activity, ligand binding and transport activity.

Figure 4 (panels A & B) summarizes the distribution of acetylation sites given in Figure 3. Nearly two thirds (64.3%) of all identified lysine acetylation sites were associated with mitochondrial proteins (Figure 4A). Cytoplasmic targets comprise about 13% of sites, whereas nuclear proteins accounted for a further 8%. Non-mitochondrial membrane associated proteins accounted for only a small fraction of total sites (5%). Notably, sarcomeric and cytoskeletal proteins account for just over 10% of lysine acetylation. Figure 4B shows the distribution of sites among major biological processes. Lipid metabolism and oxidative phosphorylation are heavily targeted by acetylation, harboring 18 and 17% of sites, respectively. Enzymes of the tri-carboxylic acid (TCA) cycle garner 12% of sites. As noted in Figure 4A, muscle contraction, as a process, is well represented with 10% of sites located within the contractile apparatus and cytoskeleton. Proteins involved in protein synthesis, metabolism and folding are prominent (8.5%), as are proteins associated with chromatin remodeling and transcription (7.7%). Finally, proteins associated with redox homeostasis or antioxidant defense, as well transporters and the enzymes of glycolysis together account for nearly 16% of acetylation sites. Proteins associated with canonical signaling pathways did not account for many sites (0.6%).

To assess the degree to which specific biological processes and molecular functions are particularly targeted by lysine acetylation, more detailed assessment of associated ontologies was performed using the Cytoscape plug-in called BINGO. Biological processes and molecular functions that are more prominent within the list than would be expected by chance are ranked according to their Benjamini & Hochberg-corrected p-values and in Table S3; Biological processes that are overrepresented relative to genomic background include metabolism, Redox sensing and contractile related ontologies. Consistent with prior work [5], specifically targeted metabolic processes include glycolysis (GO ID: 6096), fatty acid oxidation (GO ID:19395) and mitochondrial oxidative phosphorylation (GO ID: 6119). Redox sensing ontologies include response to reactive oxygen species (GO ID: 302), oxygen and reactive oxygen species metabolic process (GO ID: 6800) among others. Finally, a unique aspect of the cardiac acetylome is the enrichment of sarcomere-associated ontologies within the dataset including muscle contraction (GO ID 6936), myofibril assembly (GO ID:30239), sarcomere organization (GO ID: 45214) as well as others. The genes that define these enriched ontologies are in Table S3 (Panel 1, column G).

Figure 4C depicts the statistically-enriched ontological landscape (p<0.01) of molecular functions represented in this dataset, which falls into 3 major segments: binding activity, catalytic activity and transport functions. Among the catalytic functions, transferase, oxidoreductase, and ATPase activities predominate; oxygen-radical responsive ontologies are also represented. Ligand-binding functions are distributed among protein, ion and metabolite binding. Finally transport functions encompass acyl-carriers but primarily center around ion transport, owing to the presence of proteins such as VDAC, SERCA and RyR.

### Distribution of Acetylation Sites

Nearly 70% (165/240) of identified acetyl-proteins harbored more than one acetylation site. In Table 1, we present the top 40 most heavily acetylated proteins in our study, which account for 492 of 994 (49%) of identified sites. Again, mitochondrial processes including fatty acid oxidation, TCA cycle and oxidative phosphorylation figure prominently in this table. The histones, as expected, are also heavily acetylated. Notable, however, is the extent of acetylation on myosin heavy chain (49) sites and the thin filament regulatory protein, cardiac Troponin I (8 sites). Without further characterization, it is unclear whether these proteins constitute acetylation “hot-spots” of regulatory significance or whether they are simply among the more abundant proteins in the heart, on which one might expect to identify acetylation more often, even at low stoichiometry.

We also note that among the most heavily acetylated proteins (e.g. top 10), the distribution of assigned MS/MS spectra is often heavily weighted toward a much smaller subset of sites. For example, though myosin heavy chain acetylation was detected on 49 sites, 10 sites account for 72% of the site-counting spectra for that protein. Similarly, for Trifunctional Protein, alpha subunit (Table 1, 2^nd^ entry), 5 of the 25 acetylation sites account for 62% of the site-counting spectra. The relative intra-protein frequency of site detection among these multi-acetylated proteins is a cryptic metric. It may provide a crude first approximation of relative site occupancy. However, intrinsic peptide properties, including ionizability, fragmentation susceptibility, and affinity for the acetyl-lysine antibody, are potential confounding factors. Limitations notwithstanding, the site identification frequency on a heavily targeted protein may help prioritize the design of mutants and is, therefore, provided in Table S4.

### Mass Spectral Data is Corroborated by Anti-Acetyl-Lysine Immunoreactivity

**Figure 5:**
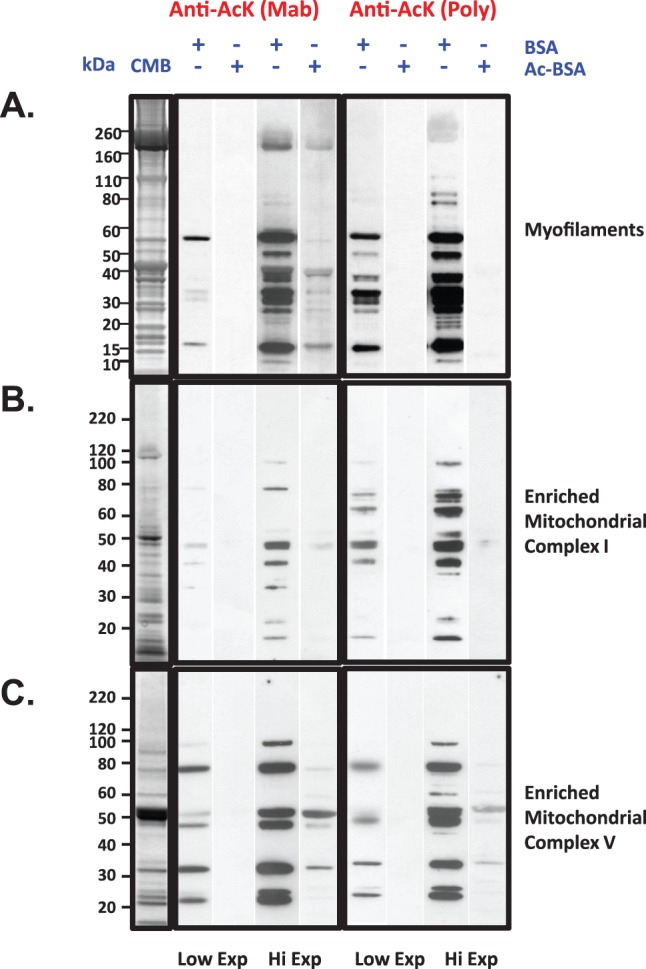
Immunodetection of Cardiac Lysine Acetylation. Panel A. Guinea pig cardiac myofilaments were prepared essentially as described by Solaro *et al.* [70]. 30 µg of protein was subjected to electrophoresis and immunoblotting with both monoclonal and polyclonal antibodies to acetylated lysine as described in the Methods section. Lanes with immunoreactive bands are juxtaposed with the corresponding exposure of a parallel control experiment conducted with >2% w/v acetylated BSA in the primary antibody incubation step. Panels B and C. Macromolecular respiratory complexes were resolved as described in the methods. Up to 20 µg of protein from the 30% w/v sucrose fraction (enriched Complex I, Panel B) and the 22.5% w/v sucrose fraction (enriched complex V, Panel C) were probed for immunoreactivity to anti-acetylated lysine as described for Panel A.

Comparison of our dataset to other global-scale proteome studies revealed 53 acetyl proteins unique to the guinea pig cardiac dataset (Table S2, panel 2). Notable within the list were myofilament proteins and calcium handling proteins. Specifically, myosin heavy chain and cardiac Troponin I were among the top 40 most heavily acetylated proteins. To assess the acetylation status of these proteins, myofilaments were prepared in the presence of lysine deacetylase inhibitors (Figure 5A). Myofilament preparations have a defined protein complement and characteristic appearance by SDS-PAGE owing to the abundant myosin heavy chain at 200 kDa and actin at 42 kDa. Acetylation status of the myofilaments was assessed by immunoblotting with polyclonal and monoclonal anti-acetylation antibodies. The two antibodies appeared to differ with respect to preferred binding targets by western blotting at low exposures. Longer exposure ultimately revealed similar acetyl-protein labeling profiles.

Acetylation of prominent mitochondrial substrates was also confirmed by sucrose density gradient enrichment of the respiratory chain complexes. Partially purified Complex I (NADH dehydrogenase; panel 5B) and Complex V (ATP synthase; panel 5C) displayed immunoreactivity toward both monoclonal and polyclonal anti acetyl-lysine antibodies on multiple subunits. The immunoreactivity of myofilaments, complex I and complex V were all diminished by performing the blots in the presence of competing acetylated BSA (>2% w/v), which confirmed the specificity of the antibodies for acetylated lysine residues.

## Discussion

### About the Model

For this proteomic study, we chose the guinea pig model since aspects of its cardiac physiology more closely approximate humans, than do rat or mouse hearts. Specifically, unlike other small animal models such as the rat, mouse, or hamster, the guinea pig has an action potential profile that displays a long plateau and a complement of ion channels and exchangers that is very similar to that of humans. In addition, the waveform of the guinea pig electrocardiogram is similar to humans [36], permitting the study of QT alterations and arrhythmias associated with heart failure. Moreover, the balance of Ca^2+^ fluxes, i.e., the relative amount of Ca^2+^ entering and leaving the cell on each heartbeat versus that released and recycled through intracellular Ca^2+^ stores is close to that of larger animals and humans [37]. In contrast, other rodents have strikingly different electrophysiological and Ca^2+^ handling properties that make it difficult to extrapolate findings to the human disease process. Other large animals, such as the rabbit, dog or cat could also provide relevant information; however, at much greater expense. Another major advantage is that the basic properties of guinea pig cardiac physiology have been extensively characterized previously, and the aortic banding model of hypertrophy and failure has also been validated in prior studies, including changes occurring in ion channels and Ca^2+^ handling that are similar to findings in human failing hearts. An additional advantage is that the computational models of the guinea pig cardiomyocytes we have developed are the most comprehensive of any species available, and the only ones that incorporate energetics and ROS metabolism [38], [39], [40].

At the outset of the project, predicted or confirmed guinea pig protein sequences were scarce in prominent protein databases (e.g. UniprotKB, NCBI Protein). However, proteomic analysis was possible using the predicted protein sequences obtained from the whole guinea pig genome. The Ensembl CavPor3.59 database contains 19,744 predicted protein sequences, enough to minimize the likelihood of failing to identify key proteins. To our knowledge this is one of few large-scale proteomic studies conducted in the guinea pig model system [41], [42]; the collected peptide data may help refine efforts to validate gene models by proteogenomics.

### The Cardiac Lysine Acetylome

The present work revealed the diverse nature of acetylation targets in the guinea pig heart and identified a large number of novel targets for which functional assessment is warranted in the future. GO analysis revealed that acetylated proteins from mitochondrial ontologies including lipid metabolism, redox balance and ATP synthesis were more prominent than expected by chance (Figure 4, Table S3). Another notable acetyl-protein identified was beta-myosin heavy chain, which contained the largest number of acetylation sites located near important catalytic regions of the myosin head. Given the expanding interest in the role of acetylation and deacetylation in aging and cardiovascular disease, the findings provide an essential primary dataset from which additional comparative studies may be launched.

#### Metabolism and respiratory chain targets

Recent work on lysine acetylation has shown that the enzymes of metabolism are prime targets from prokaryotes [5], [43] to humans [3], [6]. The cardiac acetylome shares substantial similarity to that of liver. Specifically, all of the major carbon metabolic pathways expressed in heart are targeted. Glycolysis, the pentose phosphate pathway, fatty acid oxidation, and TCA cycle are all heavily acetylated. The only notable absences among acetylated metabolic pathways are those normally found in the liver but not the heart, e.g. gluconeogenesis and the urea cycle.

Liver proteome studies showed a large number of acetylation sites attributed to mitochondrial processes [4], and given that mitochondria account for nearly 1/3 of the cellular volume in cardiomyocytes, this likely explains the observation that the lion’s share of all identified acetylation sites (64%) in our dataset. Acetylation has already been shown to inhibit the activity of complexes I [44] and II [45]. Indeed, it has recently been suggested that deacetylation of the electron transport chain, in particular the NDUFS1 subunit of Complex I and the Rieske subunit of complex III, may underlie the protection from ischemia/reperfusion injury conferred by caloric restriction, and therefore represent potential therapeutic targets [13]. Our study identified 5 acetylation sites on NDUFS1 confined primarily to its C-terminal domain, and 2 sites on Rieske subunit at K^46^ and K^101^. However, given that our gel-free strategy identified at total of 163 sites on multiple subunits of each of the respiratory complexes (Figure 3), the number of therapeutic targets may be greater than previously thought. Complex V (ATP synthase), alone, is acetylated at 68 sites. In the case of subunit d, in the stator domain of ATP synthase, acetylation is peppered along its length at 12 of 17 total lysine residues, and sequence coverage was 77% on the basis of acetylated peptides alone.

Interestingly, it has been shown that knockout of the main mitochondrial deacetylase, SIRT3, in mice, correlates with lower basal levels of ATP. Although, the detailed mechanism underlying the integrated response of metabolism to activation of deacetylation remains to be determined, the general theme of increased OxPhos, improved antioxidant activity, decreased death pathway activation and resistance to cardiac stress has been suggested [46]. These effects are presumably related to both direct modulation of metabolic enzymes by deacetylation, as well as to activation of transcriptional activators including PGC-1α and FOXO (forkhead box O transcription factor), which increase mitochondrial biogenesis and antioxidant protein expression, respectively.

We detected many previously reported acetyl-protein targets in the fatty acid oxidation pathway, TCA cycle and electron transport chain in our survey. In general, we significantly extended the number of sites modified/protein as well as the number of subunits modified per multiprotein complex. For example, for the electron transport chain, we found 29 sites on 14 subunits of complex I and 64 sites on 8 subunits of the ATP synthase. In other cases, like the A subunit of succinate dehydrogenase, acetylation at K^179^, was not detected in our study, despite identifying more sites on that subunit (cf. [45]). Acetylation of VDAC, malate dehydrogenase 2 (MDH2) and mitochondrial creatine kinase (mCK) were detected in a study of mitochondrial and cytosolic proteins from mouse hearts. Interestingly a specific peptide of VDAC was deacetylated upon feeding of mice after fasting, whereas acetylation of certain MDH peptides and one mCK peptide increased. All four of these highly regulated acetylation sites (VDAC(K^224^); MDH2(K^51^, K^156^); mCK(K^344^); mouse sequence numbering), were corroborated in the guinea pig dataset.

#### Enzymes that affect the redox environment

We note that enzymes of antioxidant defense and redox regulation are affected by acetylation. This group includes the ROS scavenger enzymes, superoxide (O_2_^.−^) dismutase 1 (SOD1) and 2 (SOD2), and the thiol protective antioxidant enzymes, thioredoxin and the peroxiredoxins. Deacetylation of mitochondrial MnSOD (SOD2), mediated by SIRT3 [47], [48], increases its O_2_^.−^ -scavenging activity and decreases overall ROS generation in response to caloric restriction [47]. Two sites whose deacetylation are deemed responsible for SOD2 activation (human K^68^ and K^122^) [48], [49] are found in the guinea pig heart. Given the that SOD2 activity has been implicated in hypertrophy and heart failure progression [50], it will be important to see whether the extent of acetylation at these sites plays a role.

Aside from the ROS scavenger pathways, proteins responsible for setting the pyridine nucleotide redox potential in mitochondria are among the primary targets of lysine acetylation in the heart. Nicotinamide nucleotide transhydrogenase (NNT) harnesses the mitochondrial proton-motive force to maintain matrix NADPH levels. Other major sources of NADPH include isocitrate dehydrogenase 2 (IDH2) and malic enzyme (ME). Both NNT and IDH2 are among the top 40 most heavily acetylated proteins (Table 1), and harbor 13 and 23 acetylation sites respectively. Thus, a coordinated response to changes in acetylation/deacetylation could include regulation of both the source of electrons required to drive the antioxidant enzymes (NADPH) and regulation of the activity of the scavenging enzymes themselves.

#### Nuclear acetylation in the heart

Nuclear acetyl-proteins were found in our myofilament-rich fraction. Among them was p300, the transcriptional co-activator with histone acetyltransferase activity. Using our global approach, we confirmed that all 9 of the autoacetylation sites, within its flexible activation loop, that were previously identified in an intensive study of bacterially overexpressed p300 [51], are also acetylated in the heart. Among the core histones that comprise the nucleosome, acetylation sites were found in isoforms of H2A, H2B, H3, H4, including, but not limited to, those summarized in a snapshot by Kazourides [52]. In addition, acetylation of the linker histone H1, known primarily as a phosphoprotein, was detected in a global study of proliferating cells by Choudhary *et al*. [3], as well as targeted studies of H1 in cells and certain mouse tissues [53], [54]. Here we confirm that Histone H1 is likewise acetylated in the guinea pig heart.

### Cardiac-Specific Lysine Acetylation Targets the Excitation-Contraction Coupling Axis

#### Calcium-handling proteins

The most unique aspect of the cardiac lysine acetylome is the observed modification of proteins involved in excitation-contraction coupling. Among them, the cardiac ryanodine receptor, RyR2, responsible for Ca^2+^ release from sarcoplasmic reticulum (SR) stores, is acetylated at K^1087^ (human K^1141^), in its cytoplasmic foot structure, specifically within a domain designated SPRY-2. The precise role of the SPRY-2 domain in RyR function is unclear, though it has been postulated that it may participate in both inter- and intra-molecular protein interactions [55].

**Figure 6:**
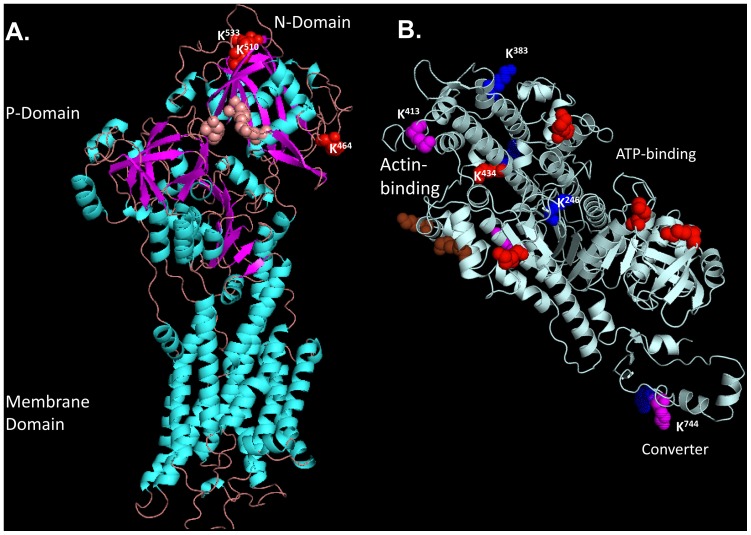
Lysine Acetylation of Two Key Proteins in EC Coupling. Panel A depicts a structural ribbon model of guinea pig SERCA2a with helices in cyan and beta strands in magenta; the membrane domain, the phosphorylation domain (P-domain) and the nucleotide binding domain (N-domain) are shown. Residues that are important for nucleotide binding are shown in salmon hue. The positions of lysine residues acetylated in our study (K^464^, K^510^, K^533^) are confined to the distal end of the cytoplasmic nucleotide-binding domain (N-domain), and are highlighted in red. Panel B depicts a model of guinea pig myosin heavy chain beta S1 head. Helices and beta strands are shown in pale cyan for simplicity. The positions of lysines acetylated in the dataset are highlighted. Magenta residues represent acetylation sites identified by the most spectra. Red residues were identified by fewer spectra. Solid blue lysine residues are sites of acetylation that are mutated in HCM. Spotted blue residues indicate positions of HCM mutation immediately adjacent to an acetylation site. Brown residues denote the positions of lysine residues found to be acetylated in previous studies by Samant *et al* [61]. Lysines highlighted in the discussion are numbered for clarity in each panel.

Sequestration of Ca^2+^ from the cytoplasm, back into the SR, is performed by the sarcoplasmic/endoplasmic reticulum Ca^2+^/ATPase, of which the SERCA2a isoform is found in the heart. Here we show that guinea pig SERCA2 is acetylated at 3 sites, all of which reside in the cytoplasmic nucleotide-binding domain (N-domain). Preliminary structural modeling of the guinea pig sequence and sites using the rabbit structure (PDB ID: 3AR4; with bound ATP, no Ca^2+^) [33] as a template (Figure 6A), shows the amino acids critical to binding of the adenine ring (i.e. E^442^, F^487^ and K^515^) [56], form a pocket that is somewhat buried. By contrast, the acetylation sites (K^464^, K^510^, K^533^) lie on the protein surface. However, both K^510^ and K^533^ do lie on the opposite end of the same beta sheet as critical residue K^515^, which begs the question of whether the structural consequences of acetylation at these surface sites might be transduced inward and thereby perturb the adenine-binding site.

### Myofilament Targets

#### Myosin and myosin light chains

Our studies show sarcomeric acetylation at the level of both the thick and thin filaments. Thick filament myosin is heavily acetylated at up to 49 sites (Table 1). Just over half of the sites map to the C-terminal tail portion that forms a coiled-coil rod with a second myosin heavy chain. The rest are distributed throughout the myosin head and neck region, some of which are modeled structurally in Figure 6B. Specifically, the most frequently identified site, K^413^, lies within the so-called hypertrophic cardiomyopathy (HCM) loop that forms part of myosin’s actin-binding interface. Indeed, several lysines acetylated in our study are found in key functional domains. For instance, K^383^ lies near the distal end of myosin where the head interacts with actin, and its mutation has been associated with HCM (K^383^N) [57]. Likewise, acetylation was also detected on K^246^, an HCM locus (K^246^Q) [58] that lies on the 6^th^ beta strand of the central beta sheet known as the transducer domain, which couples movements between the actin- and ATP-binding domain sites [59]. Interestingly, since site-directed replacement of K with Q has been used experimentally to mimic constitutive lysine acetylation (e.g. ref [60]), one might predict, conversely, that elevated acetylation at K^246^ may recapitulate the myosin and cardiac dysfunction conferred by the K^246^Q mutation. Other notable acetylation sites include K^434^ adjacent to HCM mutation site, M^435^, in the long helix at the tip of the myosin head, and K^744^ adjacent to E^743^ in the converter domain, which serves to amplify small conformational changes in the myosin head into large swings in the myosin lever arm that accompany myosin ATPase activity and drive muscle contraction. Intriguingly, whether lysine acetylation adjacent to HCM mutations might contribute to phenotypic variability observed among HCM patients has not been investigated.

The effects of lysine acetylation on myosin have been investigated recently [61]. Work by Gupta and coworkers identified the acetyltransferase, PCAF [14], and both class I and class II histone deacetylases (HDAC4 and HDAC3, respectively) [14], [61] in cardiac sarcomeres. Acetylation of myosin increased thin filament sliding velocity in *in vitro* motility assays, and levels of myosin acetylation increased when cardiac hypertrophy was induced by thoracic aortic constriction in mice. *In vitro*, myosin is acetylated by PCAF at K^549^ and K^633^, though only K^633^ was identified from cardiac myocytes cultured in the presence of deacetylase inhibitors and acetyl-CoA. Neither site was among the many identified in our study, though we would not dispute the previous acetylation site assignments, as there are substantial differences in the methodology employed. Rather, our data, taken together with prior studies of myosin acetylation, simply point to the need for rigorous site-by-site determination of *in vivo* acetylation stoichiometry.

We also show that acetylation sites are found in the in the N- and C-terminal regions of the myosin regulatory light chain (RLC), MYL2, at K^46^ and K^165^. The primary function of the myosin light chains (MYL2 and MYL3) is to bind and provide stability to the myosin lever arm. In mice, K^46^ is conserved among the atrial, ventricular, skeletal and smooth muscle isoforms, whereas K^165^ is found only in the striated muscle isoforms. K^46^ lies between D^45^ and D^48^, both of which are key Ca^2+^/Mg^2+^-coordinating residues of the EF hand domain in MYL2 [62]. Indeed, replacement of D^48^ by mutation to alanine is sufficient to ablate Ca^2+^-binding [62], and incorporation of mutated RLC into Triton X-100-skinned muscle fibers reduces isometric force production and crossbridge kinetics [63]. Therefore, given the position of K^46^ within the metal-binding loop, appreciable levels of acetylation have the potential to alter myofilament kinetics and cardiac function.

#### Thin filaments

The thin filaments consist of filamentous actin, lined with the regulatory proteins, tropomyosin, and the three proteins that comprise the troponin complex, Troponins C, I and T. In this study, each of these thin filament proteins is acetylated, and two of them, cardiac troponin I (cTnI) and tropomyosin, harbor 8 and 7 sites, respectively. In cTnI, 3 sites lie within the N-terminal half of the molecule in regions that bind Troponin T as well as the C-terminal lobe of TnC. 3 more sites, are found within the C-terminal mobile domain, near the so-called “second-actin binding site” and in the C-terminus region responsible for proper Ca^2+^-dependent tropomyosin movement over actin [64]. One of the N-terminal sites, K^36^, is a residue whose mutation causes dilated cardiomyopathy (K^36^Q) [65], [66], whereas another acetylation site, K^193^, is adjacent to R^192^, whose mutation confers restrictive cardiomyopathy (RCM, R^192^H) [65]. Given both the distribution of acetylation sites across the length of troponin I at critical sites of protein-protein interaction, and the proximity to certain HCM/RCM loci, the modification could conceivably exert effects on parameters ranging from assembly of the complex to Ca^2+^-sensitivity and cooperativity of Ca^2+^ activation. Ultimately, the extent of these effects would depend on the acetylation stoichiometry.

### Conclusion

Here, we present the first glimpse of the guinea pig cardiac lysine acetylome. The dataset may not necessarily be comprehensive, as it is subject to any biases that may arise from the specificity of the acetyl-lysine antibody chosen for affinity-peptide enrichment, though this strategy was recently used to identify as many as 15,474 sites from 16 rat tissues [67]. Another way to identify more acetyl-peptides without compromising stringent false discovery rates would be to use a cocktail of anti-acetyl-lysine antibodies from multiple vendors [68]. This is noteworthy because though key facets of cardiac EC-coupling were underrepresented in our study (e.g. ion channels of the plasma membrane), they may yet be implicated as acetyl-lysine-bearing proteins.

Finally, from the proteomic data we have extracted working hypotheses by which lysine acetylation may affect the function of several novel targets and have an impact on excitation-contraction coupling. Developing these hypotheses further, however, will require detailed assessments of acetylation site stoichiometry. Moreover, if acetylation of these targets does regulate their function, over what time scale does it occur? Indeed, the dynamics of acetylation in the heart have not yet been fully addressed, nor has the extent of crosstalk with other lysine modifications, including ubiquitination, sumoylation and methylation among others. There have been reports of lysine acetylation interfering with serine phosphorylation, particularly where the acetylated lysine is immediately N-terminally adjacent to a serine (K(Ac)S) [69]. Perusal of the acetylated peptide data in Table S1 indicates that there are 43 such examples of K(Ac)S dipeptides in our dataset that might warrant further scrutiny. These issues, among others, are particularly germane to our ongoing investigation of how global lysine acetylation may be altered in experimental models of heart failure.

## Supporting Information

Table S1 **PTM Count & Site Probability, Spectrum Report, Peptide Report.** Sheet 1 shows the sites of acetylation, the statistical probability that the acetyl group can be assigned to a specific lysine residue (in lower case k), and the the number of times that a site was identified in each of 24 LC-MS/MS runs. Sheet 2 provides spectrum data obtained for all acetylated peptides. Sheet 3 includes the data for all peptides, including unacetylated peptides that also eluted from the immunocapture resin. Sheet 4 shows the acetylated peptides in which the site of acetylation (k) lies N-terminally adjacent to serine.
(XLSX)

Table S2 **Acetyl-proteins & Sites.** Sheet 1: Additional annotation is provided for Figure 3 in the manuscript, including the numbering of orthologous lysines in human sequences. A simplified gene ontology annotation (GO SLIM) is also provided as well as the distribution of identified peptides across biological samples and subcellular fractions. Sheet 2 provides a list of the genes represented in our dataset that were not present in the studies of Choudhary *et al* [3]., Zhao *et al* [6]. or Kim *et al* [4].
(XLSX)

Table S3 **BINGO Analysis.** Sheet 1: Gene set enrichment of ontologies related to biological processes. Sheet 2: Gene set enrichment of ontologies related to molecular function. Sheet 3: Gene set enrichment of ontologies related to cellular components. These Tables include only terms deemed statistically significant (p<0.01) after Benjamini-Hochberg correction for multiple-hypothesis testing.
(XLSX)

Table S4 **Intraprotein Site Identification Frequency.** For each acetylation site identified, the number of spectra implicating that site is expressed as a percentage of the total number of spectra attributed to all acetylation sites within a given protein. This provides an indication of the relative frequency with which each site on a protein was identified. Despite confounding factors discussed in the text, it may provide a tool to help prioritize the construction of site-mutants for proteins with many acetylation sites.
(XLSX)

## References

[1] Allfrey V, Faulkner R, Mirsky AE (1964) Acetylation and methylation of histones and their possible role in the regulation of RNA synthesis. Proceedings of the National Academy of Sciences, U S A 51: 786–794. 10.1073/pnas.51.5.786 PMC300163 14172992

[2] Grunstein M (1997) Histone acetylation in chromatin structure and transcription. Nature 389: 349–352. 9311776 10.1038/38664

[3] Choudhary C, Kumar C, Gnad F, Nielsen ML, Rehman M, et al (2009) Lysine acetylation targets protein complexes and co-regulates major cellular functions. Science 325: 834–840. 19608861 10.1126/science.1175371

[4] Kim SC, Sprung R, Chen Y, Xu Y, Ball H, et al (2006) Substrate and functional diversity of lysine acetylation revealed by a proteomics survey. Molecular Cell 23: 607–618. 16916647 10.1016/j.molcel.2006.06.026

[5] Wang Q, Zhang Y, Yang C, Xiong H, Lin Y, et al (2010) Acetylation of metabolic enzymes coordinates carbon source utilization and metabolic flux. Science 327: 1004–1007. 20167787 10.1126/science.1179687 PMC4183141

[6] Zhao S, Xu W, Jiang W, Yu W, Lin Y, et al (2010) Regulation of cellular metabolism by protein lysine acetylation. Science 327: 1000–1004. 20167786 10.1126/science.1179689 PMC3232675

[7] Trivedi CM, Zhu W, Wang Q, Jia C, Kee HJ, et al (2010) Hopx and Hdac2 interact to modulate GATA4 acetylation and embryonic cardiac myocyte proliferation. Developmental Cell 19: 450–459. 20833366 10.1016/j.devcel.2010.08.012 PMC2947937

[8] Trivedi CM, Lu MM, Wang Q, Epstein JA (2008) Transgenic overexpression of HDAC3 in the heart produces increased postnatal cardiac myocyte proliferation but does not induce hypertrophy. Journal of Biological Chemistry 283: 26484–26489. 18625706 10.1074/jbc.M803686200 PMC2546558

[9] Kou CY-C, Lau SL-Y, Au K-W, Leung P-Y, Chim SS-C, et al (2010) Epigenetic regulation of neonatal cardiomyocytes differentiation. Biochemical & Biophysical Research Communications 400: 278–283. 20735989 10.1016/j.bbrc.2010.08.064

[10] Li L, Zhu J, Tian J, Liu X, Feng C (2010) A role for GCN5 in cardiomyocyte differentiation of rat mesenchymal stem cells. Molecular and Cellular Biochemistry 345: 309–316. 20835911 10.1007/s11010-010-0586-3

[11] Feng C, Zhu J, Zhao L, Lu T, Zhang W, et al (2009) Suberoylanilide hydroxamic acid promotes cardiomyocyte differentiation of rat mesenchymal stem cells. Experimental Cell Research 315: 3044–3051. 19445929 10.1016/j.yexcr.2009.05.005

[12] Backs J, Olson EN (2006) Control of cardiac growth by histone acetylation/deacetylation. Circulation Research 98: 15–24. 16397154 10.1161/01.RES.0000197782.21444.8f

[13] Shinmura K, Tamaki K, Sano M, Nakashima-Kamimura N, Wolf AM, et al (2011) Caloric restriction primes mitochondria for ischemic stress by deacetylating specific mitochondrial proteins of the electron ransport chain/novelty and significance. Circulation Research 109: 396–406. 21700931 10.1161/CIRCRESAHA.111.243097

[14] Gupta MP, Samant SA, Smith SH, Shroff SG (2008) HDAC4 and PCAF bind to cardiac sarcomeres and play a role in regulating myofilament contractile activity. Journal of Biological Chemistry 283: 10135–10146. 18250163 10.1074/jbc.M710277200 PMC2442284

[15] Colussi C, Rosati J, Straino S, Spallotta F, Berni R, et al (2011) Lysine acetylation determines dissociation from GAP junctions and lateralization of connexin 43 in normal and dystrophic heart. Proceedings of the National Academy of Sciences, USA 108: 2795–2800. 10.1073/pnas.1013124108 PMC3041095 21282606

[16] Scott I, Webster BR, Li JH, Sack MN (2012) Identification of a molecular component of the mitochondrial acetyltransferase programme: a novel role for GCN5L1. Biochemical Journal 443: 655–661. 22309213 10.1042/BJ20120118 PMC7461726

[17] Foster DB, Rucker JJ, Marban E (2008) Is Kir6.1 a subunit of mitoK_ATP_? Biochemical & Biophysical Research Communications 366: 649–656. 18068667 10.1016/j.bbrc.2007.11.154 PMC2276631

[18] Hanson BJ, Schulenberg B, Patton WF, Capaldi RA (2001) A novel subfractionation approach for mitochondrial proteins: A three-dimensional mitochondrial proteome map. Electrophoresis 22: 950–959. 11332763 10.1002/1522-2683()22:5<950::AID-ELPS950>3.0.CO;2-D

[19] Wessel D, Flugge UI (1984) A method for the quantitative recovery of protein in dilute solution in the presence of detergents and lipids. Analytical Biochemistry 138: 141–143. 6731838 10.1016/0003-2697(84)90782-6

[20] Craig R, Beavis RC (2004) TANDEM: matching proteins with tandem mass spectra. Bioinformatics 20: 1466–1467. 14976030 10.1093/bioinformatics/bth092

[21] Keller A, Nesvizhskii AI, Kolker E, Aebersold R (2002) Empirical statistical model to estimate the accuracy of peptide identifications made by MS/MS and database search. Analytical Chemistry 74: 5383–5392. 12403597 10.1021/ac025747h

[22] Nesvizhskii AI, Keller A, Kolker E, Aebersold R (2003) A statistical model for identifying proteins by tandem mass spectrometry. Analytical Chemistry 75: 4646–4658. 14632076 10.1021/ac0341261

[23] Tabb DL, Friedman DB, Ham A-JL (2006) Verification of automated peptide identifications from proteomic tandem mass spectra. Nature Protocols 1: 2213–2222. 17406459 10.1038/nprot.2006.330 PMC2819013

[24] Beausoleil SA, Villen J, Gerber SA, Rush J, Gygi SP (2006) A probability-based approach for high-throughput protein phosphorylation analysis and site localization. Nature Biotechnology 24: 1285–1292. 10.1038/nbt1240 16964243

[25] Maere S, Heymans K, Kuiper M BiNGO: a Cytoscape plugin to assess overrepresentation of Gene Ontology categories in Biological Networks. Bioinformatics 21: 3448–3449. 15972284 10.1093/bioinformatics/bti551

[26] Shannon P, Markiel A, Ozier O, Baliga NS, Wang JT, et al (2003) Cytoscape: A software environment for integrated odels of biomolecular interaction networks. Genome Research 13: 2498–2504. 14597658 10.1101/gr.1239303 PMC403769

[27] Fraenkel-Conrat H (1957) [11] Methods for investigating the essential groups for enzyme activity. Methods in Enzymology: Academic Press. 247–269.

[28] Riordan JF, Vallee BL (1972) Acetylation. In: C. H. W. Hirs SNT, editor. Methods in Enzymology: Academic Press. 494–499. 10.1016/S0076-6879(72)25045-5 23014430

[29] Arnold K, Bordoli L, Kopp Jr, Schwede T (2006) The SWISS-MODEL workspace: a web-based environment for protein structure homology modelling. Bioinformatics 22: 195–201. 16301204 10.1093/bioinformatics/bti770

[30] Bordoli L, Kiefer F, Arnold K, Benkert P, Battey J, et al (2008) Protein structure homology modeling using SWISS-MODEL workspace. Nature Protocols 4: 1–13. 10.1038/nprot.2008.197 19131951

[31] Kiefer F, Arnold K, K√°nzli M, Bordoli L, Schwede T (2009) The SWISS-MODEL Repository and associated resources. Nucleic Acids Research 37: D387–D392. 18931379 10.1093/nar/gkn750 PMC2686475

[32] Schwede T, Kopp Jr, Guex N, Peitsch MC (2003) SWISS-MODEL: an automated protein homology-modeling server. Nucleic Acids Research 31: 3381–3385. 12824332 10.1093/nar/gkg520 PMC168927

[33] Toyoshima C, Yonekura S-I, Tsueda J, Iwasawa S (2011) Trinitrophenyl derivatives bind differently from parent adenine nucleotides to Ca^2+^-ATPase in the absence of Ca^2+^ . Proceedings of the National Academy of Sciences 108: 1833–1838. 10.1073/pnas.1017659108 PMC3033254 21239683

[34] Beausoleil SA, Villen J, Gerber SA, Rush J, Gygi SP (2006) A probability-based approach for high-throughput protein phosphorylation analysis and site localization. Nat Biotech 24: 1285–1292. 10.1038/nbt1240 16964243

[35] Schwer B, Eckersdorff M, Li Y, Silva JC, Fermin D, et al (2009) Calorie restriction alters mitochondrial protein acetylation. Aging Cell 8: 604–606. 19594485 10.1111/j.1474-9726.2009.00503.x PMC2752488

[36] Suckow M, Stevens K, Wilson R (2012) The Laboratory Rabbit, Guinea Pig, Hamster and Other Rodents. London; Waltham MA: Academic Press/Elsevier.

[37] Bers D (2001) Excitation-contraction coupling and cardiac contractile force. Dordrecht; Boston: Kluwer Academic Publishers.

[38] Kembro JM, Aon MA, Winslow RL, O'Rourke B, Cortassa S (2013) Integrating mitochondrial energetics, redox and ROS metabolic networks: a two-compartment model. Biophysical Journal 104: 332–343. 23442855 10.1016/j.bpj.2012.11.3808 PMC3552263

[39] Zhou L, Aon MA, Almas T, Cortassa S, Winslow RL, et al (2010) A reaction-diffusion model of ROS-Induced ROS release in a mitochondrial network. PLoS Computational Biology 6: e1000657. 20126535 10.1371/journal.pcbi.1000657 PMC2813265

[40] Wei A-C, Aon MA, O'Rourke B, Winslow RL, Cortassa S (2011) Mitochondrial energetics, pH regulation, and ion dynamics:a computational-experimental approach. Biophysical Journal 100: 2894–2903. 21689522 10.1016/j.bpj.2011.05.027 PMC3123977

[41] Kruh NA, Troudt J, Izzo A, Prenni J, Dobos KM (2010) Portrait of a pathogen: the *mycobacterium tuberculosis* proteome *in vivo* . PLoS ONE 5: e13938. 21085642 10.1371/journal.pone.0013938 PMC2978697

[42] Giblin FJ, David LL, Wilmarth PA, Leverenz VR, Simpanya MF (2013) Shotgun proteomic analysis of S-thiolation sites of guinea pig lens nuclear crystallins following oxidative stress in vivo. Molecular Vision 19: 267–280. 23401655 PMC3566901

[43] Zhang J, Sprung R, Pei J, Tan X, Kim S, et al (2009) Lysine acetylation Is a highly abundant and evolutionarily conserved modification in Escherichia coli. Molecular & Cellular Proteomics 8: 215–225. 18723842 10.1074/mcp.M800187-MCP200 PMC2634580

[44] Ahn BH, Kim HS, Song S, Lee IH, Liu J, et al (2008) A role for the mitochondrial deacetylase Sirt3 in regulating energy homeostasis. Proceedings of the National Academy of Sciences, U S A 105: 14447–14452. 10.1073/pnas.0803790105 PMC2567183 18794531

[45] Cimen H, Han M-J, Yang Y, Tong Q, Koc H, et al (2009) Regulation of succinate dehydrogenase activity by SIRT3 in mammalian mitochondria. Biochemistry 49: 304–311. 10.1021/bi901627u PMC2826167 20000467

[46] Sack MN (2012) The role of SIRT3 in mitochondrial homeostasis and cardiac adaptation to hypertrophy and aging. Journal of molecular and cellular cardiology 52: 520–525. 22119802 10.1016/j.yjmcc.2011.11.004 PMC3294048

[47] Qiu X, Brown K, Hirschey MD, Verdin E, Chen D (2010) Calorie restriction reduces oxidative atress by SIRT3-mediated SOD2 activation. Cell Metabolism 12: 662–667. 21109198 10.1016/j.cmet.2010.11.015

[48] Chen Y, Zhang J, Lin Y, Lei Q, Guan K-L, et al (2011) Tumour suppressor SIRT3 deacetylates and activates manganese superoxide dismutase to scavenge ROS. EMBO Reports 12: 534–541. 21566644 10.1038/embor.2011.65 PMC3128277

[49] Tao R, Coleman MC, Pennington JD, Ozden O, Park S-H, et al (2010) Sirt3-mediated deacetylation of evolutionarily conserved lysine 122 regulates MnSOD Activity in response to stress. Molecular Cell 40: 893–904. 21172655 10.1016/j.molcel.2010.12.013 PMC3266626

[50] Li Y, Huang TT, Carlson EJ, Melov S, Ursell PC, et al (1995) Dilated cardiomyopathy and neonatal lethality in mutant mice lacking manganese superoxide dismutase. Nature genetics 11: 376–381. 7493016 10.1038/ng1295-376

[51] Thompson PR, Wang D, Wang L, Fulco M, Pediconi N, et al (2004) Regulation of the p300 HAT domain via a novel activation loop. Nature Structural & Molecular Biology 11: 308–315. 10.1038/nsmb740 15004546

[52] Kouzarides T (2007) SnapShot: Histone-modifying enzymes. Cell 131: 822, 822.e821–822, 822.e821. 10.1016/j.cell.2007.11.005 18022374

[53] Wisniewski JR, Zougman A, Kruger S, Mann M (2007) Mass spectrometric mapping of linker Histone H1 variants reveals multiple acetylations, methylations, and phosphorylation as well as differences between cell culture and tissue. Molecular & Cellular Proteomics 6: 72–87. 17043054 10.1074/mcp.M600255-MCP200

[54] Snijders APL, Pongdam S, Lambert SJ, Wood CM, Baldwin JP, et al (2008) Characterization of post-translational modifications of the linker histones h1 and h5 from chicken erythrocytes using mass spectrometry. Journal of Proteome Research 7: 4326–4335. 18754630 10.1021/pr800260a

[55] Tae H, Casarotto M, Dulhunty A (2009) Ubiquitous SPRY domains and their role in the skeletal type ryanodine receptor. European Biophysics Journal 39: 51–59. 19399493 10.1007/s00249-009-0455-8

[56] Toyoshima C, Inesi G (2004) Structural basis of ion pumping by Ca^2+^-ATPase of the sarcoplasmic reticulum. Annual Review of Biochemistry 73: 269–292. 10.1146/annurev.biochem.73.011303.073700 15189143

[57] Kuang S-Q, Yu J-D, Lu L, He L-M, Gong L-s, et al (1996) Identification of a novel missense mutation in the cardiac beta-myosin heavy chain gene in a chinese patient with sporadic hypertrophic cardiomyopathy. Journal of Molecular and Cellular Cardiology 28: 1879–1883. 8899546 10.1006/jmcc.1996.0180

[58] Rottbauer W, Grunig E, Brown BD, Zehelein J, Scheffold T (1996) A novel beta-myosin heavy chain mutation in a large family suffering from dilated and hypertrophic cardiomyopathy. Circulation 94: 943–943.

[59] Coureux P-D, Sweeney HL, Houdusse A (2004) Three myosin V structures delineate essential features of chemo-mechanical transduction. EMBO J 23: 4527–4537. 15510214 10.1038/sj.emboj.7600458 PMC533045

[60] Shulga N, Wilson-Smith R, Pastorino JG (2010) Sirtuin-3 deacetylation of cyclophilin D induces dissociation of hexokinase II from the mitochondria. Journal of Cell Science 123: 894–902. 20159966 10.1242/jcs.061846 PMC3189253

[61] Samant SA, Courson DS, Sundaresan NR, Pillai VB, Tan M, et al (2011) HDAC3-dependent reversible lysine acetylation of cardiac myosin heavy chain isoforms modulates their enzymatic and motor activity. Journal of Biological Chemistry 286: 5567–5577. 21177250 10.1074/jbc.M110.163865 PMC3037670

[62] Reinach FC, Nagai K, Kendrick-Jones J (1986) Site-directed mutagenesis of the regulatory light-chain Ca^2+^/Mg^2+^ binding site and its role in hybrid myosins. Nature 322: 80–83. 3523256 10.1038/322080a0

[63] Diffee GM, Patel JR, Reinach FC, Greaser ML, Moss RL (1996) Altered kinetics of contraction in skeletal muscle fibers containing a mutant myosin regulatory light chain with reduced divalent cation binding. Biophysical Journal 71: 341–350. 8804617 10.1016/S0006-3495(96)79231-7 PMC1233485

[64] Galinska A, Hatch V, Craig R, Murphy AM, Van Eyk JE, et al (2010) The C terminus of cardiac troponin I stabilizes the Ca2+-activated state of tropomyosin on actin filaments. Circulation Research 106: 705–711. 20035081 10.1161/CIRCRESAHA.109.210047 PMC2834238

[65] Tardiff JC (2011) Thin Filament Mutations. Circulation Research 108: 765–782. 21415410 10.1161/CIRCRESAHA.110.224170 PMC3075069

[66] Carballo S, Robinson P, Otway R, Fatkin D, Jongbloed JDH, et al (2009) Identification and functional characterization of cardiac troponin I as a novel disease gene in autosomal dominant dilated cardiomyopathy. Circulation Research 105: 375–382. 19590045 10.1161/CIRCRESAHA.109.196055

[67] Lundby A, Lage K, Weinert BT, Bekker-Jensen DB, Secher A, et al (2012) Proteomic analysis of lysine acetylation sites in rat tissues reveals organ specificity and subcellular patterns. Cell Reports 2: 419–431. 22902405 10.1016/j.celrep.2012.07.006 PMC4103158

[68] Shaw PG, Chaerkady R, Zhang Z, Davidson NE, Pandey A (2011) Monoclonal antibody cocktail as an enrichment tool for acetylome analysis. Analytical Chemistry 83: 3623–3626. 21466224 10.1021/ac1026176 PMC3205458

[69] Yang X-J, Seto E (2008) Lysine acetylation: codified crosstalk with other posttranslational modifications. Molecular Cell 31: 449–461. 18722172 10.1016/j.molcel.2008.07.002 PMC2551738

[70] Solaro RJ, Pang DC, Briggs FN (1971) The purification of cardiac myofibrils with Triton X-100. Biochimica et Biophysica Acta (BBA) - Bioenergetics 245: 259–262. 4332100 10.1016/0005-2728(71)90033-8

